# Writing a manuscript for publication in a peer-reviewed scientific journal: Guidance from the European Society of Clinical Pharmacy

**DOI:** 10.1007/s11096-023-01695-6

**Published:** 2024-02-08

**Authors:** Francesca Wirth, Cathal A. Cadogan, Daniela Fialová, Ankie Hazen, Monika Lutters, Vibhu Paudyal, Anita E. Weidmann, Betul Okuyan, Martin C. Henman

**Affiliations:** 1 Department of Pharmacy, Faculty of Medicine and Surgery, University of Malta, Msida, Malta; 2 School of Pharmacy and Pharmaceutical Sciences, Trinity College Dublin, Dublin, Ireland; 3 Department of Social and Clinical Pharmacy, Faculty of Pharmacy in Hradec Králové, Charles University, Hradec Králové, Czech Republic; 4 Department of Geriatrics and Gerontology, 1st Faculty of Medicine, Charles University, Prague, Czech Republic; 5 Julius Centre for Health Sciences and Primary Care, University Medical Center Utrecht, Utrecht, The Netherlands; 6 Cantonal Hospital Aarau, Aarau, Switzerland; 7 School of Pharmacy, College of Medical and Dental Sciences, Sir Robert Aitken Institute for Medical Research, University of Birmingham, Edgbaston, Birmingham, United Kingdom; 8 Department of Clinical Pharmacy, Innsbruck University, Innsbruck, Austria; 9 Department of Clinical Pharmacy, Faculty of Pharmacy, Marmara University, Istanbul, Türkiye

**Keywords:** Clinical pharmacy, Journal article, Peer review, Publishing, Research, Writing

## Abstract

Publishing in reputable peer-reviewed journals is an integral step of the clinical pharmacy research process, allowing for knowledge transfer and advancement in clinical pharmacy practice. Writing a manuscript for publication in a journal requires several careful considerations to ensure that research findings are communicated to the satisfaction of editors and reviewers, and effectively to the readers. This commentary provides a summary of the main points to consider, outlining how to: (1) select a suitable journal, (2) tailor the manuscript for the journal readership, (3) organise the content of the manuscript in line with the journal’s guidelines, and (4) manage feedback from the peer review process. This commentary reviews the steps of the writing process, identifies common pitfalls, and proposes ways to overcome them. It aims to assist both novice and established researchers in the field of clinical pharmacy to enhance the quality of writing in a research paper to maximise impact.

## Background

Clinical pharmacy research combines clinical and health services research [1]. Well-written publications derived from rigorous clinical pharmacy research studies have the potential to inform clinical decision-making and advance practice for the benefit of patients and society [1–3]. Researchers nowadays are under considerable pressure to publish for acquisition of funding, academic positions, and promotions [4–7], and publication of articles in peer-reviewed scientific journals remains the standard method of research dissemination [3, 8–10]. However, publishing in journals may be perceived as an arduous and intimidating task, with uncertainty amongst both novice and experienced researchers on how to best approach the process. Frequent questions include when, where and how to publish [6, 10]. Moreover, researchers may hesitate to pursue publishing their work in a journal due to apprehension about the peer review process and the duration of the publication process [5, 6, 11, 12]. This commentary supports the recently published Granada statements [3], and provides useful pointers to assist both early career researchers and seasoned researchers in clinical pharmacy to enhance the quality of writing in a research paper to maximise the impact.

## Choosing an appropriate journal and article format

As specified in the Granada statements, submitting a manuscript to a journal that fits the scope of the work is important to increase acceptance [3]. Authors should familiarise themselves thoroughly with the aims and scope of potential journals, and what type of manuscripts they publish. It is important to assess the following; journal scope and reach (national/international), previous published content, publishing model (open access/subscription-based), journal reputation by reviewing indexing status and journal metrics, and decision timelines [13]. While impact factor is often a key factor for author decisions on where to submit, other metrics such as citation plots, should also be considered. Researchers should aim high when selecting a journal, but should also be realistic about expectations. Even though the research may be well-executed, with robust and reliable results, choosing a very high-impact factor journal is not usually recommended, unless results are ‘paradigm-changing’ [14, 15].

Predatory (fraudulent or deceptive that claim to be legitimate) journals and publishers are usually those which undermine the conventional peer-review process for financial gain [16]. Predatory journals and publishers are becoming more prevalent; hence it is highly important for authors to identify journals and publishers which are credible. Authors should be mindful of predatory practices, such as promising to publish all submissions, publishing on payment of an article processing charge and within an unrealistic time frame, targeting of potential authors through multiple e-mails which often contain grammatical errors, and lack of transparency in the journal website regarding the peer-review process and publishing fees [16–20]. Resources such as the Committee on Publication and Ethics (COPE) [21], Open Access Scholarly Publishers Association (OASPA) [22], Directory of Open Access Journals (DOAJ) [23], and the National Library of Medicine (NLM) Catalog, may be helpful in identifying reliable journals and publishers [24].

Scholarly journals publish content in different formats, the most common being research articles (for detailed reporting of original research and primary data), short research reports (for reporting of preliminary or limited results of original research), and review articles (for critical and constructive analysis of existing published literature in a field, often identifying specific gaps or problems, and providing recommendations for future research) [25, 26]. It is important to note that not all journals offer all article formats. Once a suitable journal and article format have been selected, it is crucial to carefully review and structure the content of the manuscript according to the specific requirements in the journal’s instructions for authors, including word count, number of figures and tables, and reference style [8, 9].

## Structuring the manuscript

In scholarly writing, the Introduction, Method, Results and Discussion (IMRaD) format is typically used for ordering the manuscript [27–29], preceded by the title, abstract and keywords. The main text is followed by the conclusion, acknowledgements, references, and supporting materials [7–9, 13]. Many journals require that research papers must include, most often in a defined format, a statement putting the research in context with previous work. Editors will use this information at the first assessment stage, and peer reviewers will specifically be asked to check content and accuracy. A summary of the key points discussed in the manuscript, or impact statements, is often required. Many journals also require that submitted research articles must contain a data sharing statement, to be included at the end of the manuscript. It is advised for authors to refer to the International Committee of Medical Journal Editors (ICMJE) for recommendations on manuscript preparation and submission, including responsibilities to ensure accuracy, integrity, and originality of the work [30] (Table 1).

**Table 1:** Key points to consider when writing a journal article for publication

Select an appropriate journal, avoiding predatory journals
Follow requirements of the target journal carefully
Include an impactful title
Provide a sensibly crafted abstract
Demonstrate awareness of published literature in the field in the introduction
State the aim clearly
Describe the method accurately and adequately so that others can reproduce the work if required
Report results clearly and honestly
Discuss results in the context of what is already known and highlight what the research adds in the discussion
Acknowledge limitations and propose recommendations for future work
Provide a conclusion which is grounded in the findings presented
Ensure that the manuscript is well-formatted and proofread before submission, including tables and figures

## Writing an impactful title

The title should broadly, but adequately, reflect the content of the manuscript. It is the first exposure to the manuscript and an opportunity to attract the readers’ attention, including the editor and reviewers. A title which is impactful and that describes what has been done should be composed. The title should be concise since long titles tend to distract readers, and should be precise, informative, and easy to understand [8, 13, 31]. It is important to refer to the instructions for authors to check the type of title required by the journal since this may vary. There are three main types of titles, namely declarative (state the main findings/conclusions), descriptive (describe the topic of the article but do not reveal the main conclusions), and interrogative (introduce the subject in the form of a question) [31]. Relevant elements of the PICOS and SPIDER concepts should be applied [32], mentioning the study design where appropriate, and not including the name of the country, except when reporting a country-wide survey. Technical jargon and abbreviations should be avoided as much as possible.

## The abstract

The abstract also represents the first impression that an article will make to prospective readers, including the editor and reviewers, hence should be drafted meticulously. Decisions to proceed to the peer review process are frequently based on the clarity of information presented in the abstract. Well-written abstracts also interest reputable reviewers, since the abstract is seen before deciding to accept or decline a review invitation from the journal. The readers may be researchers and potential authors who will cite the article, or may not be researchers but are interested in the topic, hence an effective abstract is key to attract a wider readership. The abstract may be structured or unstructured in accordance with the journal requirements (refer to instructions for authors of the specific journal). Journal formatting requirements should be followed when writing the abstract, particularly with respect to word count. It is crucial to be succinct and accurate in writing, providing a comprehensible summary of the study. Authors should provide a clear and concise aim, method including study design, setting and population, and ensure that the results (key findings) presented in the abstract (and the manuscript) match the aim. Conclusions/interpretations must be supported by the study findings. It is important to avoid using jargon, uncommon abbreviations, and references in the abstract [8, 13, 31, 33, 34].

## Selecting suitable keywords

It is very important to select appropriate keywords for manuscript indexing so that the research can be retrieved in searches for other researchers to use and cite [3]. The authors’ instructions should be checked for the number of keywords to be included. Words with a broad meaning and words already included in the title should if possible be avoided. Some journals require that keywords selected are not those from the journal name. Abbreviations which are not broadly used should be avoided. Clinical pharmacy researchers are responsible for using standardised and consistent terminology, explicitly existing Medical Subject Headings (MeSH) terms, especially in titles and abstracts, to improve article visibility, as emphasised in the Granada statements [3, 35–38].

## Introduction section and review of literature

In the introduction section, a compelling and concise account of *why* the topic is important and useful within the field of clinical pharmacy, what is known about the topic and the research gap, the scientific rationale, and innovative aspects of the study, should be clearly provided. Originality needs to be justified and demonstrated particularly for journals targeting an international readership. Authors should demonstrate awareness of seminal publications in the field, incorporating recent literature and any systematic reviews. In addition, authors should provide both an international and national perspective, and refrain from giving a historical account. The information presented should guide the readers to the aims/objectives, which are included at the end of the introduction. A tip is to avoid over-exaggerated claims or expressions such as “novel,” “first time,” and “first ever”, except when this is really the case [2, 8, 9, 13, 34, 39].

## Method section

The method section should provide a transparent, sufficiently detailed, and reproducible description of *how* the study was conducted to allow replication. Established reporting guidelines from the EQUATOR Network should be followed when formatting a research paper (e.g. COREQ for qualitative research, TIDieR to describe interventions) [40]. Justification of the method selected, study setting, sampling, inclusion and exclusion criteria, development and psychometric evaluation of research instruments, data collection procedure, and data analysis approaches, must be clearly described. Details of the study protocol including validation could be published in a separate article, especially if word count is an issue. Any previously published protocols or research instruments used should be referred to, however details of established methods need not be repeated, and references/supporting materials should be used. It is also of utmost importance to add a statement to confirm that the research was conducted in accordance with the relevant ethics committee/institutional review board, and that participant consent was obtained as applicable [8, 13, 33, 41]. The method section is crucial for reviewers in appraising the work, and incomplete or inaccurate descriptions could result in the manuscript being rejected [42].

## Results section

The results section describes *what* the research has found. Only key research findings should be presented; however, it is important to remember that most journals allow inclusion of supplementary material for additional data to reinforce the conclusion. The results should be presented clearly and accurately, should link to the aims/objectives and generally follow the same order with respect to outcomes as described in the methods section. It is advisable to divide the results into sub-headings to keep results of the same type together, such as demographics, primary and secondary outcomes, and to follow a logical flow. Authors should take advantage of providing results in tables and figures, ensuring that they are cross-referenced consecutively in the text, but avoiding duplication. Each table and figure must be self-explanatory, with accompanying titles, legends, and explanation of abbreviations that are clearly written and understandable. When presenting tables and figures, authors should; not clutter them with too much data, use well-selected scales, add data labels, think about appropriate axis label size, select legible font type, and size, and include clear symbols and data sets that are easy to distinguish. The title of a table should be included above the table, and for figures, the title should be included below the figure. Authors should limit inclusion of very long tables if possible; these may be included as supplementary material. Reporting of statistical data should follow a standard approach (e.g. mean and standard deviation to report normally distributed data, median and interpercentile range to report skewed data, confidence interval, p-values, significance level). Authors should use the International System of Units of measurement, two significant figures when reporting numbers unless more precision is necessary, and avoid reporting percentages for very small samples. Discussing the findings in the results section is a common error, and interpretations should be reserved for the discussion, with reference to other studies [8, 13, 34].

## Discussion and conclusion section

In the discussion, authors should respond to *what* the results mean and *how* the work advances the field of clinical pharmacy. A manuscript is often rejected if the discussion is weak [42]. Authors should commence by providing a clear and grounded summary of the key findings to ensure that focus is maintained, and addressing the study aims/objectives. This is followed by interpretation, and not reiteration, of the presented results in the context of published literature. Results should be related to those of similar studies, attempting to explain why similar or contradictory results were obtained. Tips to consider include avoiding statements that go beyond what the results can support, and avoiding sudden introduction of new terms or ideas. It is possible to speculate on possible interpretations, however these should be rooted in fact. Authors should indicate the study’s strengths without overemphasising, discuss the implications to clinical practice, and put forward recommendations for future work/research. The study’s strengths and limitations, and how they impact the generalisability/transferability of findings should be discussed.

A clear, concise, and convincing conclusion that corresponds to the study’s objectives and results, and that reinforces the significance of the research and implications for practice should be presented. If trivial statements are included, reviewers will find it difficult to critically analyse the work and whether it merits publication in the journal. The results may not be generalisable/transferable to other study populations, so words such as ‘may’ should be used when extrapolating results [8, 9, 13, 34, 42].

## Other statements and declarations

Many journals require specifying how each author contributed to the study design and/or writing of the manuscript, justifying authorship. All authors must approve the final version of the manuscript. Relevant conflicts of interest the authors may have should be disclosed, and persons who have contributed to the manuscript but not to the extent to qualify for authorship, including data collectors, collaborators, study participants and proof-readers, should be duly thanked. It is cordial to check that those being acknowledged agree to be named in the paper. It is important to disclose funding sources, including any grant or fellowship [8, 29, 34].

## References section

Although formatting of references is nowadays easier due to available software (e.g. EndNote), there are typically more errors in the presentation of in-text citations and reference list than in any other part of the manuscript [42]. It is important to follow the referencing style of the journal and to cite all scientific publications on which the work is based. However, the manuscript should not be inflated with too many references. Recent publications should be prioritised and citing articles from predatory journals should be avoided. Self-citations and personal communications should be minimised. Unpublished work should only be cited if it contributes significantly to the manuscript. Spelling of author names, year of publication, usage of ‘et al.,’ punctuation, and whether all references are included should be checked [8, 34].

## Submission

Authors should be mindful that content is essential and use of language and presentation are critical, hence proofreading prior to submission is crucial. Most journals provide a checklist including specific requirements for different types of articles. Table 2 provides a checklist for revision and editing before manuscript submission. A manuscript should not be submitted to more than one journal concurrently.

**Table 2:** Final checklist before submission of an article to a journal

Is the language clear and precise? Where possible have article proofread by a fluent English (or the chosen language) speaker
Are there smooth transitions between sections?
Is the text in a logical order and well-structured?
Are all abbreviations used in the article explained?
Are the tables and figures in the right order?
Are the references and in-text citations in accordance with the journal’s author instructions?
Perform a final word count check
Is a cover letter to the editor prepared? The letter should put the study in context, explain why the research is of importance to the journal’s audience and why it should be considered for publication in the journal. The letter should also contain a statement that the manuscript has not been submitted elsewhere in similar form, should state that all authors have contributed significantly to the publication and that all authors are aware of the submission and agree with it
Is the title page comprehensive according to instructions for authors?

## Responding to peer review feedback

Peer review feedback is a central component of the publication process and it is common to receive minor or major revisions. It is reasonable to feel disappointed upon receiving peer review feedback, however it is crucial not to respond to comments impulsively. Authors should take adequate time to review the comments, discuss with co-authors/project team and formulate comprehensive responses, acknowledging the reviewers’ insight, and clearly highlighting any amendments made. As an author it is important to also prepare for possible rejection, and this does not mean that the manuscript has no value. Various papers that have resulted in important translation of knowledge were not accepted to the first journal to which they were submitted, hence it is important to take the peer review feedback on board to improve quality of the manuscript and to try another journal [2, 10, 12, 13, 34]. Furthermore, clinical pharmacy researchers can facilitate the efficiency of the publication process by being more proactive in becoming involved as peer reviewers, as highlighted in the Granada statements [3].

## Concluding thoughts

A well-written manuscript enables readers, especially reviewers and editors, to easily grasp the scientific significance. Writing a good manuscript is not easy, and there is no secret formula for success. Yet, publishing is a very rewarding endeavour. Successful publishing requires preparation, perseverance, diligence and learning from disappointments.

Following publication of guidance about writing a successful grant application [43], this guidance was also prepared by the Research Committee of ESCP as a part of ESCP’s commitment towards “disseminating clinical pharmacy research findings”. Tips for success in the publication process are to start early and include a publication strategy in the study protocol, choose an appropriate journal, strictly observe the guidelines and requirements for authors of the selected journal, and to follow the recommendations provided in this commentary.

Research dissemination is a responsibility for all pharmacists, including clinical pharmacists, as a foundation for new research and the application of findings [44]. Clinical pharmacy researchers should provide publications that advance knowledge and understanding. They have an obligation towards responsible research reporting, adopting research integrity and ensuring that publications are clear, accurate, complete, and balanced, avoiding misleading, selective, or ambiguous reporting [29, 45].
