# Correction: Writing a manuscript for publication in a peer-reviewed scientific journal: Guidance from the European Society of Clinical Pharmacy

**DOI:** 10.1007/s11096-024-01712-2

**Published:** 2024-03-02

**Authors:** Francesca Wirth, Cathal A. Cadogan, Daniela Fialová, Ankie Hazen, Monika Lutters, Vibhu Paudyal, Anita E. Weidmann, Betul Okuyan, Martin C. Henman

**Affiliations:** 1 Department of Pharmacy, Faculty of Medicine and Surgery, University of Malta, Msida, Malta; 2 School of Pharmacy and Pharmaceutical Sciences, Trinity College Dublin, Dublin, Ireland; 3 Department of Social and Clinical Pharmacy, Faculty of Pharmacy in Hradec Králové, Charles University, Hradec Králové, Czech Republic; 4 Department of Geriatrics and Gerontology, 1st Faculty of Medicine, Charles University, Prague, Czech Republic; 5 Julius Centre for Health Sciences and Primary Care, University Medical Center Utrecht, Utrecht, The Netherlands; 6 Cantonal Hospital Aarau, Aarau, Switzerland; 7 School of Pharmacy, College of Medical and Dental Sciences, Sir Robert Aitken Institute for Medical Research, University of Birmingham, Edgbaston, Birmingham, UK; 8 Department of Clinical Pharmacy, Innsbruck University, Innsbruck, Austria; 9 Department of Clinical Pharmacy, Faculty of Pharmacy, Marmara University, Istanbul, Türkiye

**Correction to: International Journal of Clinical Pharmacy** 10.1007/s11096-023-01695-6

In this article, the sentence ‘publishing without an article processing charge’ is corrected as ‘publishing on payment of an article processing charge’ under the section ‘Choosing an appropriate journal and article format’.

The original article has been corrected.

